# Effects of Lipid Metabolism-Related Genes PTGIS and HRASLS on Phenotype, Prognosis, and Tumor Immunity in Lung Squamous Cell Carcinoma

**DOI:** 10.1155/2023/6811625

**Published:** 2023-01-17

**Authors:** Kai Lei, Ruihao Liang, Binghua Tan, Lin Li, Yingcheng Lyu, Kexi Wang, Wenjian Wang, Kefeng Wang, Xueting Hu, Duoguang Wu, Huayue Lin, Minghui Wang

**Affiliations:** ^1^Guangdong Provincial Key Laboratory of Malignant Tumor Epigenetics and Gene Regulation, Sun Yat-sen Memorial Hospital, Sun Yat-sen University, Guangzhou, China; ^2^Department of Thoracic Surgery, Sun Yat-sen Memorial Hospital, Sun Yat-sen University, Guangzhou, China; ^3^Department of Thoracic Surgery, The First Affiliated Hospital of USTC, Division of Life Sciences and Medicine, University of Science and Technology of China, China; ^4^Breast Tumor Center, Sun Yat-sen Memorial Hospital, Sun Yat-sen University, Guangzhou, China

## Abstract

**Background:**

Lipid metabolism reprogramming played an important role in cancer occurrence, development, and immune regulation. The aim of this study was to identify and validate lipid metabolism-related genes (LMRGs) associated with the phenotype, prognosis, and immunological characteristics of lung squamous cell carcinoma (LUSC).

**Methods:**

In the TCGA cohort, bioinformatics and survival analysis were used to identify lipid metabolism-related differentially expressed genes (DEGs) associated with the prognosis of LUSC. PTGIS/HRASLS knockdown and overexpression effects on the LUSC phenotype were analyzed in vitro experiments. Based on the expression distribution of PTGIS/HRASLS, LUSC patients were divided into two clusters by consensus clustering. Clinical information, prognosis, immune infiltration, expression of immune checkpoints, and tumor mutation burden (TMB) level were compared between the TCGA and GSE4573 cohorts. The genes related to clustering and tumor immunity were screened by weighted gene coexpression network analysis (WGCNA), and the target module genes were analyzed by functional enrichment analysis, protein-protein interaction (PPI) analysis, and immune correlation analysis.

**Results:**

191 lipid metabolism-related DEGs were identified, of which 5 genes were independent prognostic genes of LUSC. PTGIS/HRASLS were most closely related to LUSC prognosis and immunity. RT-qPCR, western blot (WB) analysis, and immunohistochemistry (IHC) showed that the expression of PTGIS was low in LUSC, while HRASLS was high. Functionally, PTGIS promoted LUSC proliferation, migration, and invasion, while HRASLS inhibited LUSC proliferation, migration, and invasion. The two clusters' expression and distribution of PTGIS/HRASLS had the opposite trend. Cluster 1 was associated with lower pathological staging (pT, pN, and pTNM stages), better prognosis, stronger immune infiltration, higher expression of immune checkpoints, and higher TMB level than cluster 2. WGCNA found that 28 genes including CD4 and IL10RA were related to the expression of PTGIS/HRASLS and tumor immune infiltration. PTGIS/HRASLS in the GSE4573 cohort had the same effect on LUSC prognosis and tumor immunity as the TCGA cohort.

**Conclusions:**

PTGIS and HRASLS can be used as new therapeutic targets for LUSC as well as biomarkers for prognosis and tumor immunity, which has positive significance for guiding the immunotherapy of LUSC.

## 1. Introduction

According to the global cancer statistics in 2020, lung cancer ranked second in incidence and first in mortality [1]. Lung cancer was divided into small-cell lung cancer (SCLC) and non-small-cell lung cancer (NSCLC). NSCLC accounted for about 80-85% of lung cancer, and lung squamous cell carcinoma (LUSC) accounted for about 20-30% of NSCLC [2]. The 5-year survival rate for LUSC was only about 10% due to the lack of specific clinical manifestations in the early stage [3, 4]. LUSC was characterized by a low fusion rate of the anaplastic lymphoma kinase (ALK) gene and a low gene mutation rate of epidermal growth factor receptor (EGFR), which were about 1.5%-2.5% and 2.7%, respectively [5, 6]. Therefore, only a few LUSC patients with genetic mutations were suitable for targeted therapy. Currently, the treatment of advanced lung cancer has entered the era of immunotherapy represented by immune checkpoint inhibitors (ICIs). The immune contexture of cancer was closely related to the prognosis [7] and the efficacy of tumor immunotherapy [8, 9]. Tian et al. [10] suggested that LUSC was superior to lung adenocarcinoma (LUAD) in immunotherapy, which may be related to the mutation rate of the carcinogenic driving gene, TMB level, PD-L1 expression, and tumor-infiltrating lymphocytes in LUSC. Patients who respond to immunotherapy were most likely to achieve high-quality long-term survival [11], but inefficiency remains a thorny issue [12]. Therefore, the discovery of novel biomarkers that predict tumor prognosis and immunotherapy efficacy to select potential immunotherapy beneficiaries is a key issue in the LUSC immunotherapy field.

Abnormal lipid metabolism was an important feature of tumor metabolic reprogramming. The tumor microenvironment (TME) was hypoxic, acidic, and nutrient-deficient, which led to the metabolic reconstitution of tumor cells and adjacent stromal cells and promoted tumor cell survival, proliferation, and metastasis. Tumor cells were characterized by excessive intake of fat and cholesterol [13] while increasing new fat production [14], which was essential for membrane biosynthesis and signaling molecules. Lipid metabolism reprogramming existed in colon cancer, breast cancer, lung cancer, and prostate cancer, and the abnormal regulation of lipid metabolism in cancer cells was closely related to the occurrence and development of tumors [15, 16]. FASN (fatty acid synthase) was highly expressed in colon cancer [17], breast cancer [18], and renal cell carcinoma [19] and was associated with poor prognosis, tumor recurrence, and drug resistance. Some studies [20, 21] showed that FASN was highly expressed in NSCLC cells and promoted metastasis and cisplatin resistance of NSCLC cells. Lv et al. [22] examined plasma lipid profiles of healthy individuals and patients with different subtypes of lung cancer and found significant changes in a large number of lipoprotein-related genes in combination with data from large-scale genomic screening. Therefore, the role of LMRGs in LUSC is still worthy of further study.

Lipid metabolism reprogramming was a hallmark of malignancy and occurs in tumor cells and TME [23]. A common metabolic change in the TME was lipid accumulation associated with immune dysfunction. Xu et al. [24] have shown that the TME contained a large number of oxidized fat molecules, which could inhibit the ability of killer T cells (CD8 T cells) to kill cancer cells. Lim et al. [25] found that when SREBPs (sterol-regulator-element-binding proteins) were lost in regulatory T cells, the proportion of CD4 and CD8 T cells in TME increased, tumor growth was inhibited, and the efficacy of anti-PD1 therapy was enhanced. LMRG signature has been reported to predict the prognosis and immune characteristics of colorectal adenocarcinoma [26], osteosarcoma [27], and LUAD [28]. However, whether LMRGs can be used as biomarkers of LUSC prognosis and tumor immunity has not been reported.

To more fully understand the role of LMRG in LUSC phenotype, prognosis, and tumor immunity, we identified and validated LMRGs associated with LUSC prognosis and immunity in TCGA and GSE4573 cohorts. Furthermore, we verified the expression levels of PTGIS/HRASLS in LUSC cell lines and tissues and evaluated the role of PTGIS/HRASLS in LUSC by vitro experiments. Our work provided clues for finding novel biomarkers related to lipid metabolism in LUSC phenotype, prognosis, tumor immune infiltration, and the efficacy of tumor immunotherapy, which will be beneficial to the clinical decision-making of individualized immunotherapy in LUSC.

## 2. Materials and Methods

### 2.1. Data Acquisition

The gene set containing 742 LMRGs was obtained from the “metabolism of lipids” pathway of the Reactome pathway database (https://reactome.org/) (Supplementary Table 1). The transcriptome data (FPKM) and clinicopathological data of 498 LUSC samples and 288 normal lung tissue samples for difference analysis were derived from the TCGA TARGET GTEx dataset in the University of California Santa Cruz Xena platform (UCSC Xena; https://xena.ucsc.edu/) (Supplementary Table 2). The FPKM of all transcriptome data was transformed by log_2_(FPKM+1) for further analysis. 394 LUSC samples with complete clinical information were included in the follow-up study (Supplementary Table 3). GSE4573 cohort containing 130 LUSC samples was downloaded from the Gene Expression Omnibus database (GEO; https://www.ncbi.nlm.nih.gov/gds/) (Supplementary Table 4). Simple nucleotide variation (SNV) data of 409 LUSC cases was downloaded from The Cancer Genome Atlas database (TCGA; https://cancergenome.nih.gov/). TCGA cohort (*n* = 394) was used as the training set and GSE4573 (*n* = 130) as the validation set, and the clinical characteristics of the two cohorts were compared (Table 1).

### 2.2. Identification and Functional Enrichment Analysis of DEGs

The principal component analysis (PCA) was used to detect the clustering effect of LUSC samples from the TCGA dataset and normal lung tissue samples from the GTEx dataset. The expression matrix (FPKM) containing 498 LUSC samples and 288 normal lung tissue samples was compared with R package “limma” to obtain DEGs. The filtering criteria were *p* value < 0.05 and |log2FC| > 1. The somatic mutation landscape of DEGs in 409 LUSC samples was obtained by using the R package “maftools.” Gene Ontology (GO) and Kyoto Encyclopedia of Genes and Genomes (KEGG) enrichment analyses were performed on DEGs using the R package “clusterProfiler.”

### 2.3. Determination of Independent Prognostic Genes

We first used the univariate Cox regression analysis to screen DEGs associated with LUSC prognosis. LASSO regression analysis was then performed using the R package “glmnet” to eliminate genes with similar prognostic values. Finally, the multivariate Cox regression analysis was used to determine independent prognostic genes in LUSC. The Kaplan-Meier survival analysis was performed with the R package “survival” to study the effect of independent prognostic genes on the overall survival (OS) of LUSC.

### 2.4. Consensus Clustering of TCGA Cohort

ESTIMATE analysis (ESTIMATEScore, ImmuneScore, StromalScore, and TumorPurity) was performed for each sample using the R package “ESTIMATE” to assess the ratio of immune cells to stromal cells [29]. The correlations between 5 independent prognostic genes and 4 ESTIMATE indexes were calculated, and the genes associated with prognosis and tumor immunity were selected for follow-up analysis. The expression levels of PTGIS and HRASLS in LUSC and normal lung tissues were compared, and the correlation between them was calculated. The expression data of independent prognostic genes PTGIS and HRASLS was extracted, and consensus clustering was performed using the R software package “ConsensusClusterPlus” [30]. All samples were divided into two clusters, and the survival of the two clusters was analyzed.

### 2.5. Comparison of Immune Infiltration between Two Clusters

To better understand the functional enrichment differences between two clusters, gene set enrichment analysis (GSEA) was performed for all DEGs using the R package “clusterProfiler” [31]. We used ESTIMATE to evaluate the overall strength of tumor immunity, CIBERSORT to evaluate the infiltration ratio of 22 kinds of immune cells [32], and R package “GSVA” for the single-sample gene set enrichment analysis (ssGSEA) to evaluate the expression of 28 kinds of immune cells [33, 34].

### 2.6. Weighted Gene Coexpression Network Analysis (WGCNA)

To better characterize the DEGs of two clusters, we used the R package “WGCNA” to perform a WGCNA of the DEGs that met the requirements (*p* value < 0.05 and |log2FC| > 0.8) and to identify the coexpressed genes and modules [35]. Firstly, the samples were clustered and the abnormal samples were eliminated. Then, the optimal soft threshold was calculated as 9, the minimum number of module genes was set as 30, and 7 modules were obtained. To pick out modules related to both lipid metabolism and immunity, we calculated the correlation between each module and traits (Clustering, ESTIMATEScore, ImmuneScore, StromalScore, and TumorPurity). The genes in the module that met the criteria (|MM| (module membership) > 0.9 and |GS| (gene significance) > 0.7) were selected for subsequent analysis.

### 2.7. Collection of LUSC Samples

We collected 30 pairs of LUSC and paired tumor-adjacent normal lung tissues that underwent surgery at Sun Yat-sen Memorial Hospital of Sun Yat-sen University from 2015 to 2018. All the above have been informed consent and approved by the Medical Ethics Committee of Sun Yat-sen Memorial Hospital (SYSKY-2022-050-01).

### 2.8. Cell Culture

Two human LUSC cell lines (NCI-H226 and SK-MES-1) and one human normal lung epithelial cell line (BEAS-2B) were from the Shanghai Institutes for Biological Science, China. All cells were cultured in the 1640 (Gibco, Carlsbad, CA, USA) medium with 10% fetal bovine plasma (Gibco) and 100 U/ml streptomycins and penicillin (HyClone, Logan, UT, USA) at 37°C in a humidified atmosphere with 5% CO_2_. The cell culture medium was changed every 2 days. When nearly 80% fused, the cells were digested and passed by 0.25% trypsin. The analysis was carried out after 3-5 generations.

### 2.9. Real-Time Quantitative PCR

Total RNA was extracted using TRIzol reagent (Invitrogen, Carlsbad, CA, USA). Simply, 1 ml TRIzol reagent was used to lysis 50-100 mg tissue or 5 − 10 × 10^6^ cells. RNA was extracted, precipitated, and washed with chloroform, isopropanol, and 75% ethanol. RNA precipitation was dissolved in 30 *μ*l deionized water, and the RNA concentration was determined for subsequent analysis. cDNA was synthesized using the HiScript II Q RT SuperMix for qPCR (Vazyme, Nanjing, China). The cDNA was then analyzed by RT-qPCR using AceQ qPCR SYBR Green Master Mix (without ROX) (Vazyme) according to the manufacturer's protocol. Briefly, the RT-qPCR reaction process was divided into predenaturation (95°C for 30 s; one cycle), amplification (95°C for 10 s and 60°C for 30 s; forty cycles), and melting (95°C for 15 s, 60°C for 60 s, and 95°C for 15 s; one cycle). The expression of target transcripts was normalized to the GAPDH internal control, and relative changes in gene expression were determined using the 2^−*ΔΔ*CT^ method.

The primers for PTGIS are 5′-CTGTTGGGCGATGCTACAGAA-3′ (forward) and 5′-GCCTCAATTCCGTAAAGAGTCA-3′ (reverse), HRASLS are 5′-TGCTTCAGTTTGAACTACCCTG-3′ (forward) and 5′-GCCCAGTGCTGATAGCCAG-3′ (reverse), and GAPDH are 5′-GGAGCGAGATCCCTCCAAAAT-3′ (forward) and 5′-GGCTGTTGTCATACTTCTCATGG-3′ (reverse).

### 2.10. Western Blot (WB) Analysis

Human normal lung epithelial cells (BEAS-2B) and human LUSC cells (NCI-H226 and SK-MES-1) were lysed with RIPA buffer (CWBIO, Beijing, China) containing 1% phosphatase and protease inhibitor. The protein concentration of the sample was determined by the BCA protein quantity kit (Beyotime, Shanghai, China). 20 *μ*g of protein lysates was separated by sodium dodecyl sulfate-polyacrylamide gel electrophoresis (SDS-PAGE) and transferred to the PVDF membrane. The membrane was sealed with 5% bovine serum albumin solution at room temperature for 1 h, and the closed membrane was incubated with primary antibodies specific for PTGIS (diluted 1 : 1000, Immunoway, Newark, Delaware, USA), HRASLS (diluted 1 : 1000, Immunoway), and *β*-actin (diluted 1 : 10000, Immunoway) at 4°C overnight and then incubated with goat anti-rabbit secondary antibody for 1 h. Signals were detected with image acquisition using the enhanced chemiluminescence (ECL) reagent (Vazyme) and Optimax X-ray Film Processor (Protec, Germany).

### 2.11. Immunohistochemistry

The procedure of the immunohistochemical experiment and the scoring method of staining were mentioned earlier [36]. Two independent observers evaluated the immunostaining degree of the target protein. The scores of staining intensity and range were high positive (3+), positive (2+), low positive (1+), and negative (0). We used ImageJ software to calculate the target protein's integral optical density (IOD). Primary antibodies against PTGIS (Immunoway) and HRASLS (ImmunoWay) were used.

### 2.12. RNA Interference Analysis and Plasmid Construction

Control short interfering RNAs (siRNAs) and siRNAs targeting PTGIS/HRASLS were purchased from GenePharma (Shanghai, China). The siRNA sequences used in RNA interference analysis are listed in Table 2. Empty plasmids and overexpression plasmids of PTGIS/HRASLS were purchased from GenePharma (Shanghai, China). Transfections of siRNA and plasmid were performed using the Lipofectamine 3000 Transfection Reagent (Thermo Fisher, Scientific, Waltham, MA, USA) according to the manufacturer's protocol.

### 2.13. 5-Ethynyl-2′-deoxyuridine (EdU) Staining

An EdU kit (BeyoClick™ EdU Cell Proliferation Kit with Alexa Fluor 555, Beyotime) was adopted to inquire about the cell proliferation ability. Cells were seeded in 24-well plates with a density of 5 × 10^4^ cells per well. Subsequently, cells were incubated with EdU for 2 h, fixed with 4% paraformaldehyde for 15 min, and permeated with 0.3% Triton X-100 for another 10 min. The cells were incubated with the Click Reaction Mixture for 30 min at room temperature in a dark place and then incubated with Hoechst 33342 for 10 min. Then, the results were visualized by a fluorescence microscope.

### 2.14. Cell Migration and Invasion Assays

The cell migration or invasion assays were performed using 24-well plates inserted by an 8 *μ*m pore size transwell filter insert (Corning, New York, USA) with or without precoated diluted Matrigel (Becton Dickinson, Franklin Lakes, NJ, USA). 6 × 10^4^ LUSC cells with the serum-free medium were placed into the upper chamber, and medium containing 10% FBS was added into the bottom chamber subsequently. After incubation at 37°C for 24 h (migration) or 48 h (invasion), cells on the underside of the membrane were immobilized and stained with crystal violet (Beyotime). Then, penetrated cells were counted under a microscope and photographed.

### 2.15. Statistical Analysis

Statistical analysis was based on R software version 4.1.1 (R Foundation for Statistical Computing, Vienna, Austria), Statistical Product Service Solutions software version 26.0 (IBM Corporation, Armonk, NY, USA), and GraphPad Prism software version 7.0 (GraphPad Software, La Jolla, CA, USA). All classification variables were described by quantity (percentage), and the chi-square test was used to compare the two sets of data (use Fisher's exact test if necessary). All measurement data were described in the median (quartile), and the two sets of data were compared by *t*-test (Mann–Whitney *U* test if necessary). The Kaplan-Meier method was used for survival analysis and the log-rank test was used for analysis.

## 3. Results

### 3.1. Identification and Mutation Landscapes of Lipid Metabolism-Related DEGs

**Figure 1:**
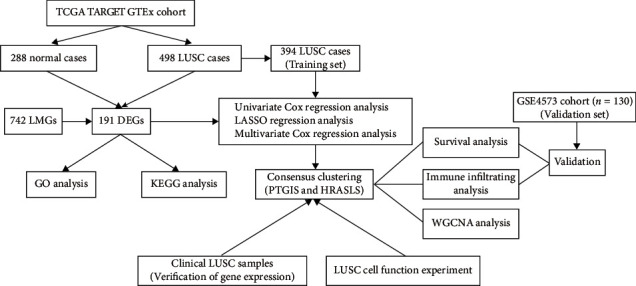
The workflow chart for this study.

**Figure 2:**
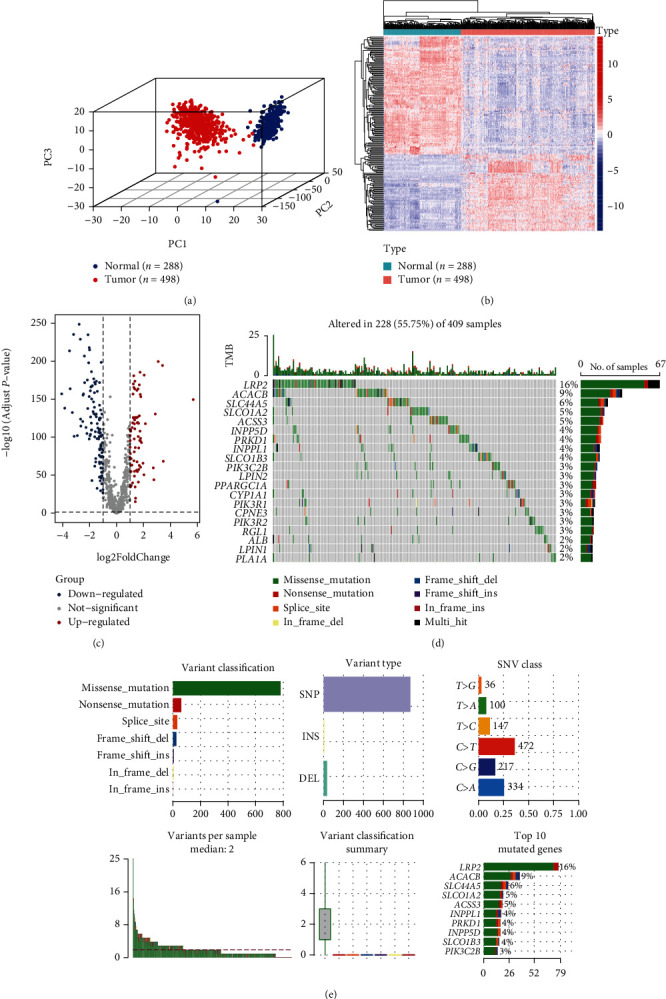
Identification and mutation landscapes of lipid metabolism-related DEGs. (a) The 3D plot of principal component analysis (PCA) between the TCGA dataset (tumor = 498) and the GTEx dataset (normal = 288). (b, c) The heatmap and volcano plot of 191 lipid metabolism-related DEGs (the filtering criteria were adjusted *p* value < 0.05 and |log2FC| > 1). (d, e) The mutation frequency and classification of 191 lipid metabolism-related DEGs in LUSC.

The workflow chart of this study is shown in Figure 1. 3D PCA plot showed that 498 LUSC samples from the TCGA dataset and 288 normal lung tissue samples from the GTEx dataset were significantly independent (Figure 2(a)). 191 lipid metabolism-related DEGs were identified by using a screening threshold of |log2FC| > 1 and *p* value < 0.05, including 116 downregulated and 75 upregulated genes (Figures 2(b) and 2(c)). We then summarized the incidence of somatic mutations in 191 DEGs in LUSC. Genetic mutations were found in 228 (55.75%) of 409 LUSC patients (Figures 2(d) and 2(e)).  Figure 2(d) shows the mutation landscape of DEGs with the top 20 mutation rates in LUSC. The missense mutation was the highest classification of variation. Single nucleotide polymorphism (SNP) was the most common type of mutation, and C>T was the single nucleotide variation (SNV) type with the highest incidence (Figure 2(e)).

### 3.2. Enrichment Analysis of DEGs

**Figure 3:**
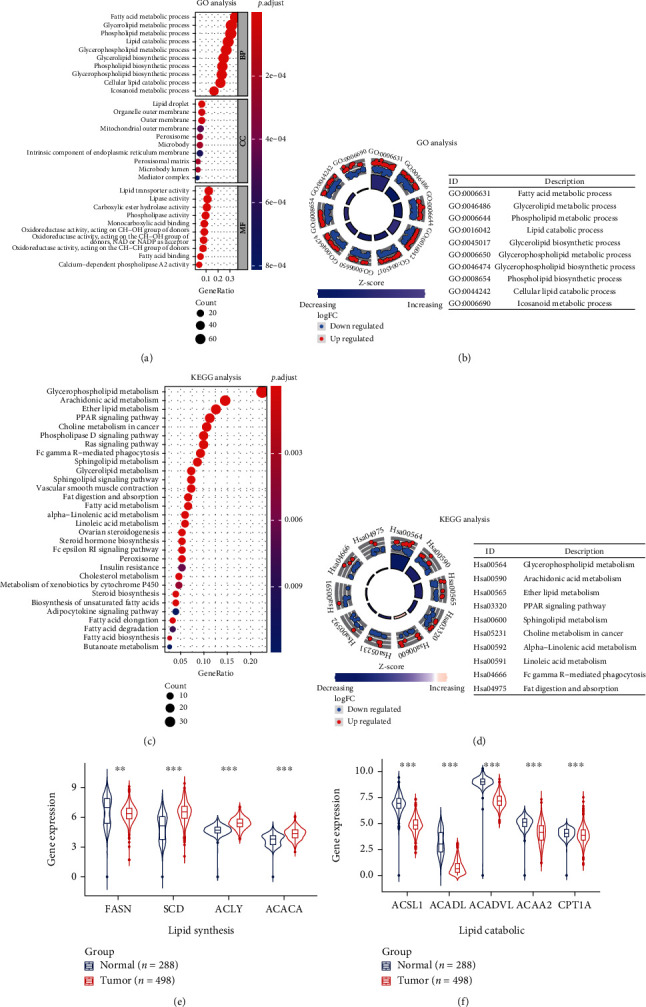
The functional enrichment analysis of 191 DEGs. (a, b) GO enrichment analysis 191 DEGs. (c, d) KEGG analysis of 191 DEGs. (e, f) The expression of key genes in lipid synthesis and lipid decomposition pathway in LUSC and normal lung tissues. ^∗∗^*p* < 0.01 and ^∗∗∗^*p* < 0.001.

GO analysis showed that these 191 DEGs were mainly involved in the fatty acid metabolic process, glycerolipid metabolic process, and phospholipid metabolic process (Figures 3(a) and 3(b)). The pathways enriched by KEGG analysis included glycerophospholipid metabolism, arachidonic acid metabolism, and ether lipid metabolism (Figures 3(c) and 3(d)). Moreover, there were significant differences in the expression of representative genes in lipid synthesis (Figure 3(e)), lipid catabolic (Figure 3(f)), and lipid uptake (Supplementary Figure 1) in LUSC compared with normal lung tissues. These results indicated the importance of lipid metabolism in LUSC.

### 3.3. Identification of Prognostic Genes Related to Lipid Metabolism in the TCGA Cohort

**Figure 4:**
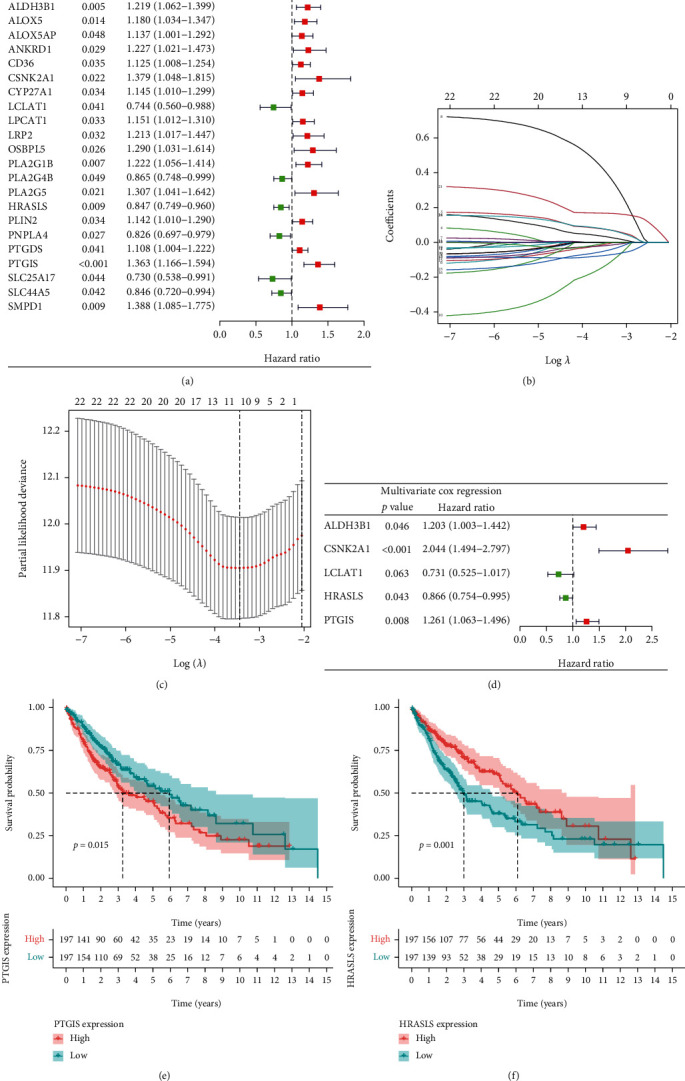
Identification of independent prognostic genes for LUSC. (a) Forest map of differentially expressed LMRGs related to LUSC survival, analyzed by the univariate Cox regression. (b) LASSO coefficient spectrum of 25 genes in LUSC. Generate a coefficient distribution map for a logarithmic (*λ*) sequence. (c) Selecting the best parameters for LUSC in the LASSO model (*λ*). (d) Forest map of independent prognostic genes in LUSC, analyzed by the multivariate Cox regression. (e) The Kaplan-Meier survival curve of PTGIS. (f) The Kaplan-Meier survival curve of HRASLS.

We used the univariate Cox regression, LASSO regression, and multivariate Cox regression analysis to screen DEGs associated with the prognosis of LUSC. 24 LMRGs related to the prognosis of LUSC were screened by the univariate Cox regression analysis (*p* value < 0.05) (Figure 4(a)). Then, these genes were involved in LASSO regression analysis, and 10 genes with the best *λ* value were identified (Figures 4(b) and 4(c)). Based on the genes produced by LASSO regression analysis, 5 independent prognostic genes of LUSC were identified by the multivariate Cox regression analysis (Figure 4(d)). The Kaplan-Meier survival analysis examined the relationship between the expression of 5 independent prognostic genes and OS in LUSC (Figures 4(e) and 4(f) and Supplementary Figure 2), in which the high expression of PTGIS (Figure 4(e)) and the low expression of HRASLS (Figure 4(f)) were associated with poor prognosis in LUSC.

### 3.4. Verification of the Expression of PTGIS and HRASLS in Cell Lines and Tissues

**Figure 5:**
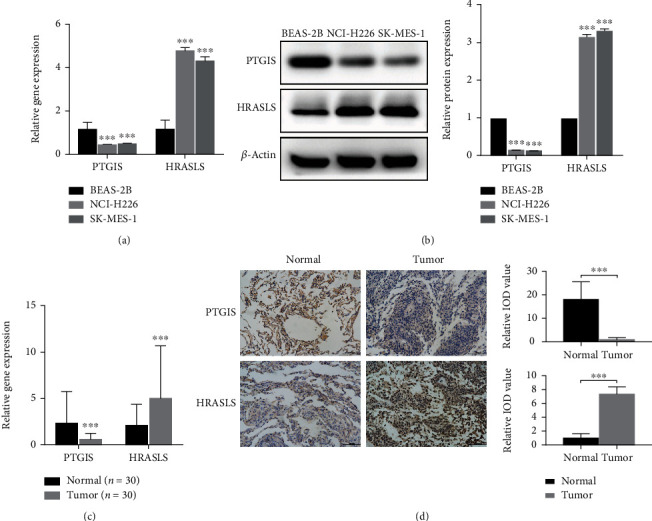
Verification of the expression of PTGIS and HRASLS in LUSC cells and tissues. (a) Detection of mRNA relative expression of PTGIS and HRASLS in LUSC cell lines by RT-qPCR. (b) Detection of protein expression of PTGIS and HRASLS in LUSC cell lines by WB analysis. (c) Detection of mRNA relative expression of PTGIS and HRASLS in LUSC and paired tumor-adjacent normal tissues by RT-qPCR. (d) Detection of protein expression of PTGIS and HRASLS in LUSC tissues and tumor-adjacent normal tissues by IHC. ^∗∗∗^*p* < 0.001.

RT-qPCR showed that the expression of PTGIS was low in LUSC cell lines and tissues, while HRASLS was high (Figures 5(a) and 5(c)). WB analysis showed that the protein level of PTGIS in LUSC cell lines (NCI-H226 and SK-MES-1) was significantly lower than that in lung epithelial cell line (BEAS-2B), and HRASLS was significantly higher (Figure 5(b)). Next, we used IHC to detect the protein expression of PTGIS and HRASLS in LUSC and paired tumor-adjacent normal lung tissues and found that the protein level of PTGIS was low in LUSC and HRASLS was high (Figure 5(d)).

### 3.5. PTGIS and HRASLS Affect LUSC Proliferation, Migration, and Invasion In Vitro

**Figure 6:**
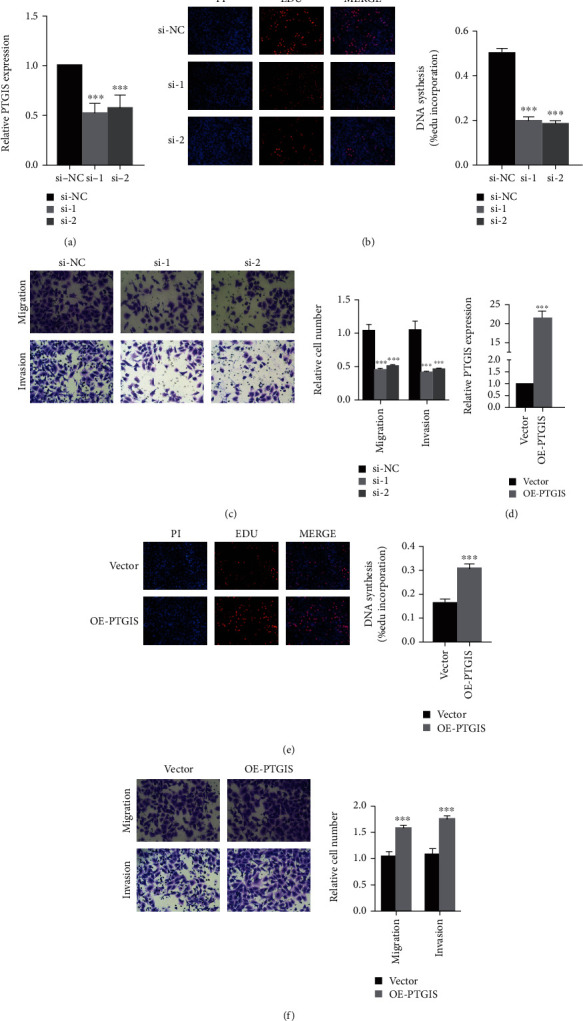
PTGIS promotes LUSC proliferation, migration, and invasion. (a) The mRNA expression of PTGIS in the SK-MES-1 cell line transfected with siRNAs or si-NC was measured by qRT-PCR. (d) The overexpression plasmid of PTGIS or the control vector was transfected into the SK-MES-1 cell line, and the mRNA expression of PTGIS was measured by qRT-PCR. (b, e) Representative images of EdU assay after PTGIS knockdown (b) and PTGIS overexpression (e) in SK-MES-1 cells. (c, f) Representative images of transwell assay after PTGIS knockdown (c) and PTGIS overexpression (f) in SK-MES-1 cells. ^∗∗∗^*p* < 0.001.

**Figure 7:**
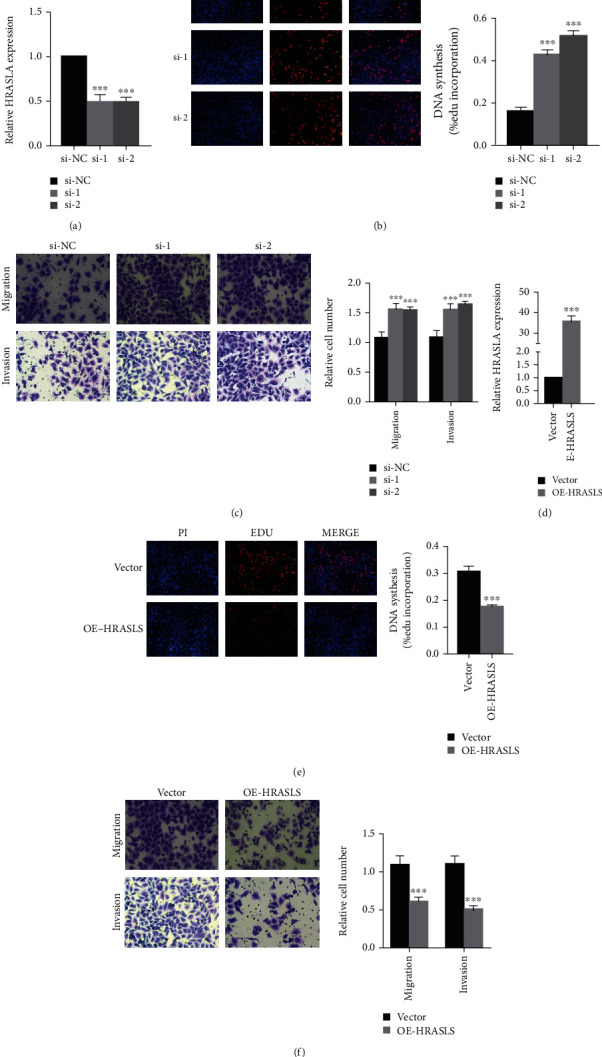
HRASLS inhibits LUSC proliferation, migration, and invasion. (a) The mRNA expression of HRASLS in the SK-MES-1 cell line transfected with siRNAs or si-NC was measured by qRT-PCR. (d) The overexpression plasmid of HRASLS or the control vector was transfected in the SK-MES-1 cell line, and the mRNA expression of HRASLS was measured by qRT-PCR. (b, e) Representative images of EdU assay after HRASLS knockout (b) and HRASLS overexpression (e) in SK-MES-1 cells. (c, f) Representative images of transwell assay after HRASLS knockout (c) and HRASLS overexpression (f) in SK-MES-1 cells. ^∗∗∗^*p* < 0.001.

To clarify the role of PTGIS and HRASLS in LUSC, we performed cell experiments in the LUSC cell line (SK-MES-1). Depletion of PTGIS and HRASLS with siRNA resulted in a significant knockdown in PTGIS (Figure 6(a)) and HRASLS (Figure 7(a)) levels. PTGIS knockout significantly inhibited LUSC cell proliferation, migration, and invasion (Figures 6(b) and 6(c)), while HRASLS knockout significantly promoted LUSC cell proliferation, migration, and invasion (Figures 7(b) and 7(c)). PTGIS and HRASLS expression plasmids resulted in significant overexpression of PTGIS (Figure 6(d)) and HRASLS (Figure 7(d)) levels. PTGIS overexpression promoted LUSC cell proliferation, migration, and invasion (Figures 6(e) and 6(f)), while HRASLS overexpression inhibited LUSC cell proliferation, migration, and invasion (Figures 7(e) and 7(f)). Taken together, these results suggested that PTGIS and HRASLS played an important role in LUSC phenotype.

### 3.6. Consensus Clustering of LUSC Based on PTGIS and HRASLS

**Figure 8:**
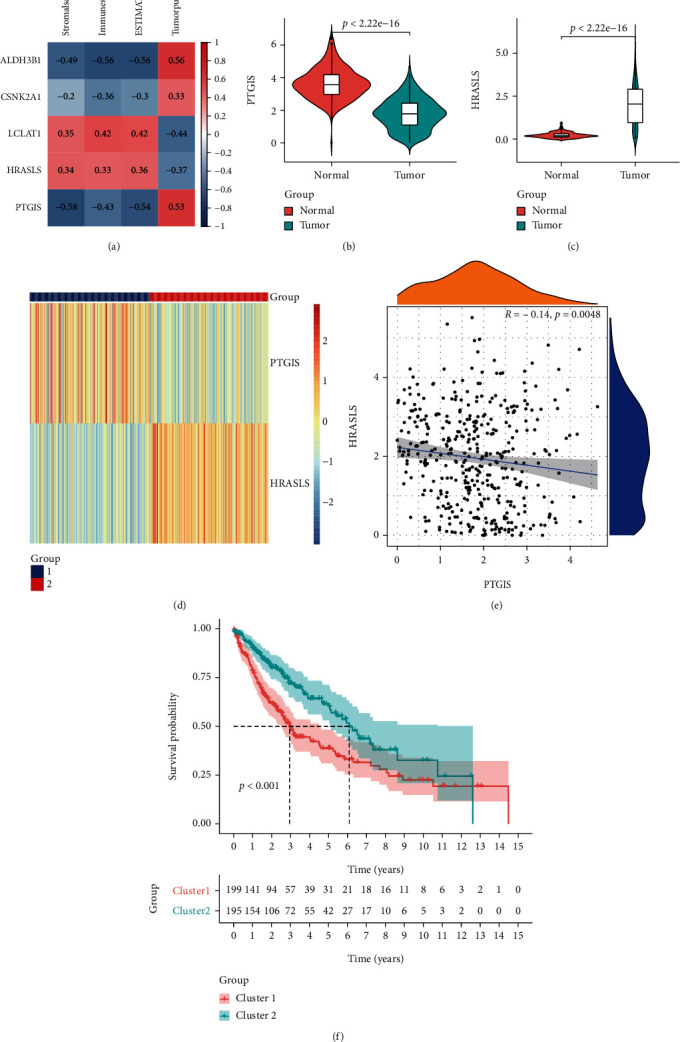
PTGIS and HRASLS were associated with the prognosis and immune score in the TCGA-LUSC cohort. (a) Association between LUSC independent prognostic genes and results of ESTIMATE. (b) The mRNA expression of PTGIS in LUSC and normal lung tissues. (c) The mRNA expression of HRASLS in LUSC and normal lung tissues. (d) Consensus clustering based on the expression distribution of PTGIS and HRASLS divided the TCGA cohort into two clusters. (e) Association between PTGIS and HRASLS expression. (f) The Kaplan-Meier survival curve of two clusters.

To explore the role of lipid metabolism in LUSC tumor immunity, we evaluated the correlation between the expression of independent prognostic genes and ESTIMATE indices in LUSC (Figure 8(a)). Considering the correlation with both OS and ESTIMATE indices, PTGIS and HRASLS were included in the follow-up analysis. We compared the expression of the two genes in the TCGA cohort and found that the expression of PTGIS in tumor tissue was lower than that in normal tissue (Figure 8(b)), while HRASLS was higher (Figure 8(c)). Next, we performed consensus clustering of 394 LUSC patients according to the expression distribution of PTGIS and HRASLS and divided the samples into two clusters (Figure 8(d) and Supplementary Figure 3). The consensus clustering heatmap showed that the expression of PTGIS was high and HRASLS was low in cluster 1 (*n* = 199), while the expression of PTGIS was low and HRASLS was high in cluster 2 (*n* = 195). Because the two genes showed opposite trends in the two clusters, the Spearman correlation between PTGIS and HRASLS was studied, and it was found that there was a weak negative correlation between them (*R* = −0.14, *p* = 0.0048) (Figure 8(e)). We compared the differences in clinical features between the two clusters and found that cluster 1 had higher pT, pN, and pTNM stages than cluster 2 (Table 3). In addition, the prognosis of cluster 2 was significantly better than that of cluster 1 (Figure 8(f)). These results suggested that PTGIS and HRASLS divided the TCGA cohort into two molecular subtypes with different characteristics.

### 3.7. Comparison of Immune Characteristics between Two Clusters

**Figure 9:**
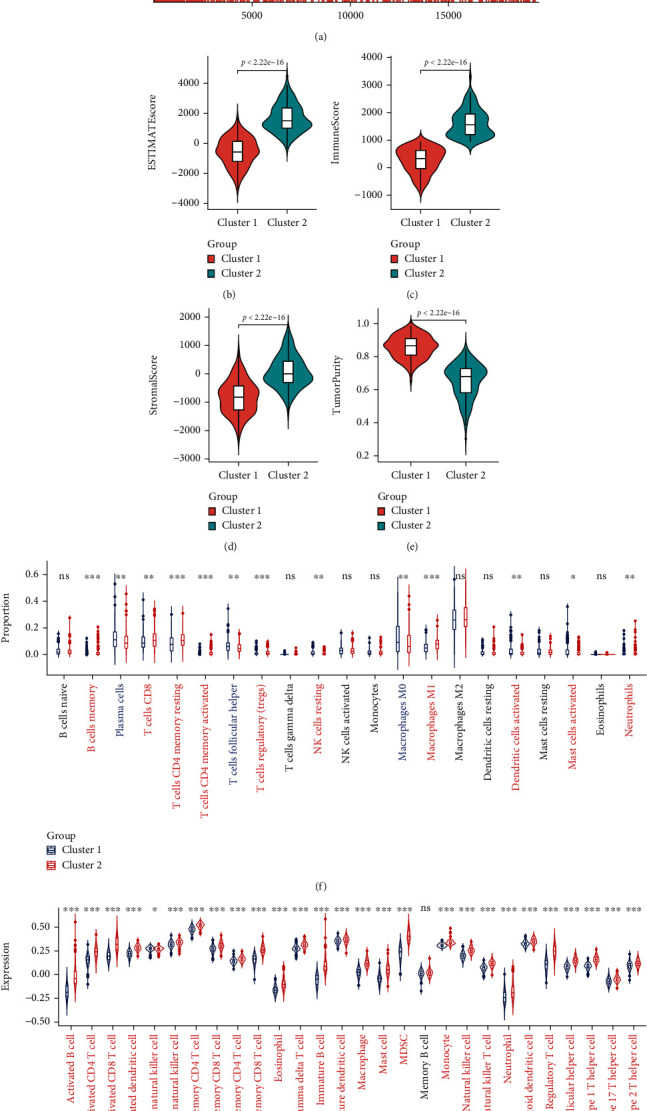
Comparison of immune characteristics between two clusters. (a) Gene set enrichment analysis (GSEA) between two clusters. Comparison of ESTIMATEScore (b), ImmuneScore (c), StromalScore (d), TumorPurity (e), the proportion of 22 immune cells (f), and expression of 28 immune cells (g) between two clusters. ns: no significance. ^∗^*p* < 0.05, ^∗∗^*p* < 0.01, and ^∗∗∗^*p* < 0.001.

To understand the functional enrichment differences between the two clusters, we included all the DEGs of the two clusters in the GSEA analysis. We have found many important pathways related to immunity in the enrichment analysis of the MSigDB Collection (c5.all.v7.4.symbols.gmt), including activation of the immune response, acute inflammation response, and adaptive immune response, and these pathways were enhanced in cluster 2 (Figure 9(a)). Then, we compared the immune infiltration of the two clusters using ESTIMATE, CIBERSORT, and ssGSEA. ESTIMATE showed that the ESTIMATEScore, ImmuneScore, and StromalScore of cluster 2 were higher (Figures 9(b)–9(d)), but TumorPurity was lower (Figure 9(e)). The infiltration heatmap of 22 immune cells showed that the proportion of M2 macrophages in LUSC was significantly higher than that of other types of immune cells (Supplementary Figure 4). CIBERSORT showed a higher proportion of CD8 T cells and M1 macrophages in cluster 2 (Figure 9(f)). ssGSEA showed that the expression of 27 immune cells of cluster 2 was higher (such as activated B cells, activated CD4 T cells, activated CD8 T cells, and natural killer cells) (Figure 9(g)). These results showed that the immune cell infiltration of cluster 2 was stronger than that of cluster 1.

### 3.8. Comparison of Gene Mutation and Evaluation of Immunotherapy Sensitivity between Two Clusters

**Figure 10:**
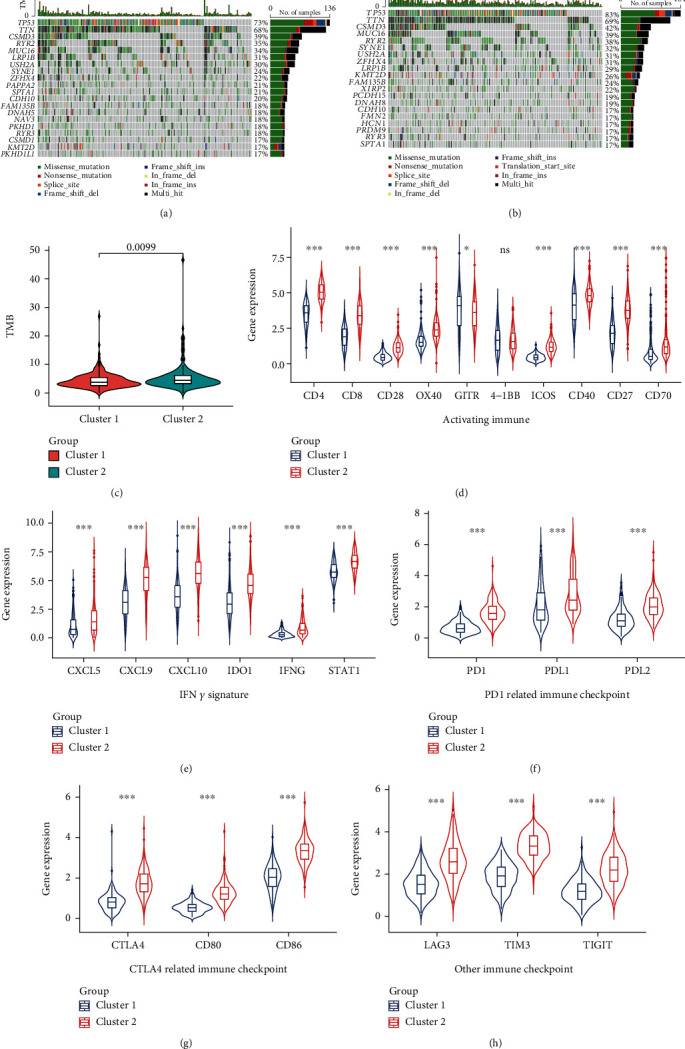
Comparison of the gene mutation landscape and the expression of immunomodulatory targets between two clusters. Mutational landscapes of cluster 1 (a) and cluster 2 (b). (c) Comparison of the tumor mutation burden (TMB) level between the two clusters. The expression levels of key genes in activating immune (d) and IFN *γ* signature (e) pathways were compared between the two clusters. The expression levels of PD1-related (PD1, PDL1, and PDL2) (f) and CTLA4-related (CTLA4, CD80, and CD86) (g) and other (LAG3, TIM3, and TIGIT) (h) immune checkpoints were compared between the two clusters. ns: no significance. ^∗^*p* < 0.05 and ^∗∗∗^*p* < 0.001.

The TMB level affected tumor immune infiltration and the efficacy of immunotherapy [10]. Therefore, we evaluated the somatic mutations of the two clusters. Figures 10(a) and 10(b) show the mutation landscapes of the two clusters, and the TMB level of cluster 2 was higher than that of cluster 1 (Figure 10(c)). This indicated that cluster 2 may be more abundant in tumor immune infiltration and more sensitive to tumor immunotherapy. Therefore, we first compared the expression of common immunoregulatory markers in the two clusters and found that the expression levels of immune activation and INF *γ* signaling pathway markers in cluster 2 were significantly higher than those in cluster 1 (Figures 10(d) and 10(e)). Then, we compared the expression of immune checkpoints between the two clusters to evaluate the sensitivity of LUSC to immunotherapy and found that the expression of PD1-related immune checkpoints (PD1, PDL1, and PDL2) (Figure 10(f)), CTLA4-related immune checkpoints (CTLA4, CD80, and CD86) (Figure 10(g)), and other reported immune checkpoints (LAG3, TIM3, and TIGIT) (Figure 10(h)) in cluster 2 were significantly higher than those in cluster 1.

### 3.9. Identification of Module Genes Related to Clustering and Immunity in Weighted Gene Coexpression Network Analysis (WGCNA)

**Figure 11:**
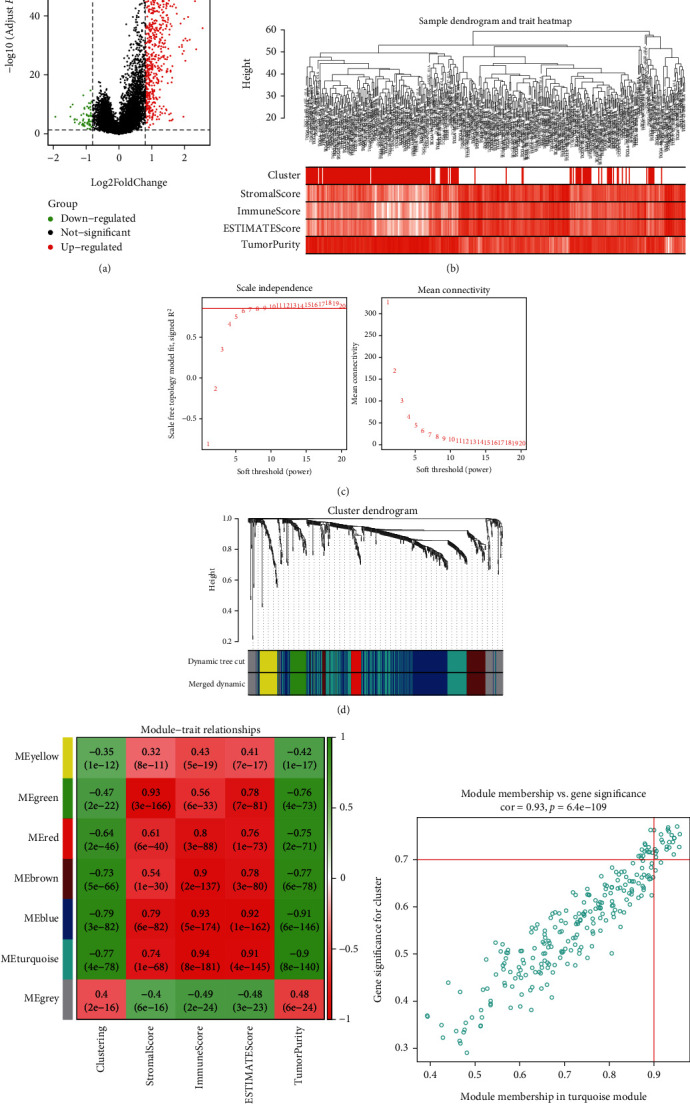
Identification of module genes related to clustering and immunity in weighted gene coexpression network analysis (WGCNA). (a) The volcano plot of DEGs (the filtering criteria were adjusted *p* value < 0.05 and |log2FC| > 0.8). (b) Sample dendrogram and trait heatmap based on two clusters of DEGs and immune characteristics in the TCGA cohort. (c) The scale-free fitting index of soft threshold power (*β*), and 9 was the most suitable power value. (d) Gene dendrogram and module colors. (e) The correlation heatmap of module eigengenes, clustering, and ESTIMATE results. (f) Scatter plot of module eigengenes in the turquoise module (the filtering criteria were |MM| > 0.9 and |GS| > 0.7).

Through the screening thresholds of |log2FC| > 0.8 and *p* value < 0.05, we identified 763 DEGs (including 697 upregulated genes and 66 downregulated genes) (Figure 11(a)). The expression matrix containing 394 LUSC samples and 763 DEGs was included in WGCNA. The sample cluster analysis eliminated 8 abnormal samples and retained 386 samples (Supplementary Figure 5). The heatmap of clustering and immune infiltration score is shown in Figure 11(b). We chose 9 as the best soft threshold (Figure 11(c)) and 30 as the minimum number of genes for each coexpression network module and obtained 7 network expression modules (Figure 11(d)). To identify modules related to clustering and immunity, we analyzed the correlation between modules and traits (Figure 11(e)). The turquoise module has a strong correlation with clustering (*r* = −0.77, *p* = 4 × 10^−78^) and immunity (*r* = 0.94, *p* = 8 × 10^−181^), so it will be used as the object of follow-up research. Finally, based on |MM| > 0.9 and |GS| > 0.7, we obtained 28 hub genes (CD53, SLA, ARHGAP15, SASH3, EVI2B, GIMAP4, SELPLG, CYTH4, PTPRC, NCKAP1L, WAS, RCSD1, IL10RA, CD74, IRF8, TNFRSF1B, BTK, SNX20, APBB1IP, CD37, DOCK2, CD4, GIMAP7, LILRB1, IL16, DOCK8, CORO1A, and BIN2) from the turquoise coexpression network module (Figure 11(f)).

### 3.10. Enrichment Analysis of Hub Genes and Their Relationship with Tumor Immune Infiltration

**Figure 12:**
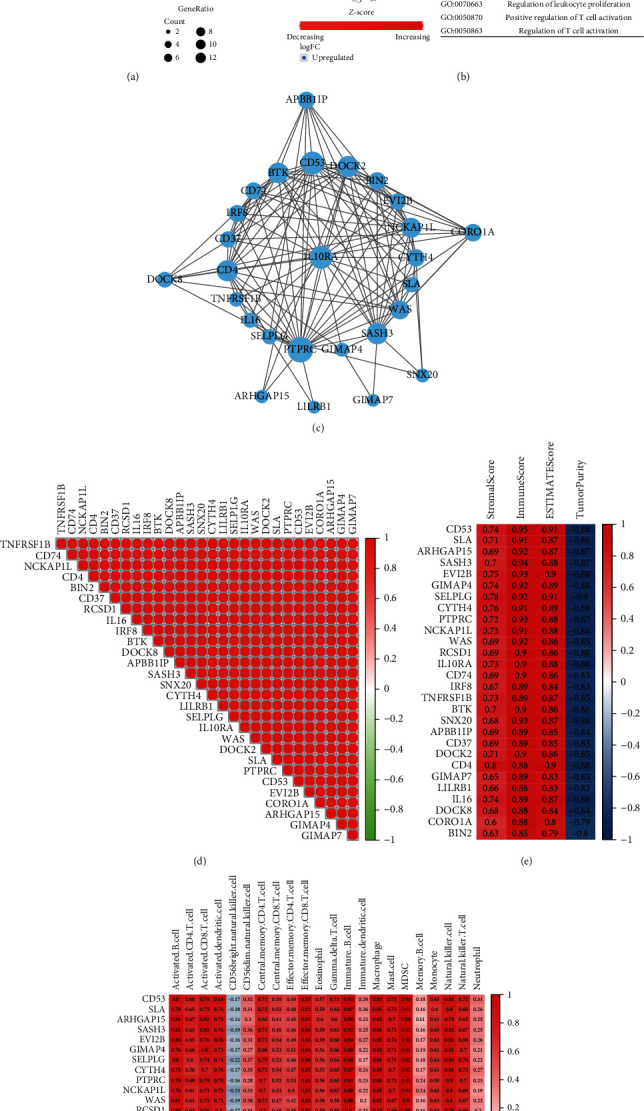
Analysis of 28 hub genes. (a, b) GO enrichment analysis of 28 hub genes. (c) The protein-protein interaction (PPI) network of 28 hub genes. (d) The correlation heatmap of 28 hub genes. (e) The correlation heatmap between hub genes and results of ESTIMATE. (f) The correlation heatmap between hub genes and expression of immune cells (ssGSEA).

We conducted GO enrichment analysis of 28 hub genes obtained by WGCNA and found that the most important GO project was the activation and proliferation of immune cells (Figure 12(a)), and these processes were enhanced (Figure 12(b)). PPI analysis showed that IL10RA was in the center of the hub gene interaction network (Figure 12(c)), and there was a strong positive correlation between these genes (Figure 12(d)). The Spearman correlation analysis of the hub genes and tumor immune infiltration analysis (ESTIMATE and ssGSEA) showed that all hub genes were significantly associated with tumor immunity (Figures 12(e) and 12(f)).

### 3.11. GEO Validation of PTGIS and HRASLS in Evaluating Prognosis and Tumor Immunity of LUSC

**Figure 13:**
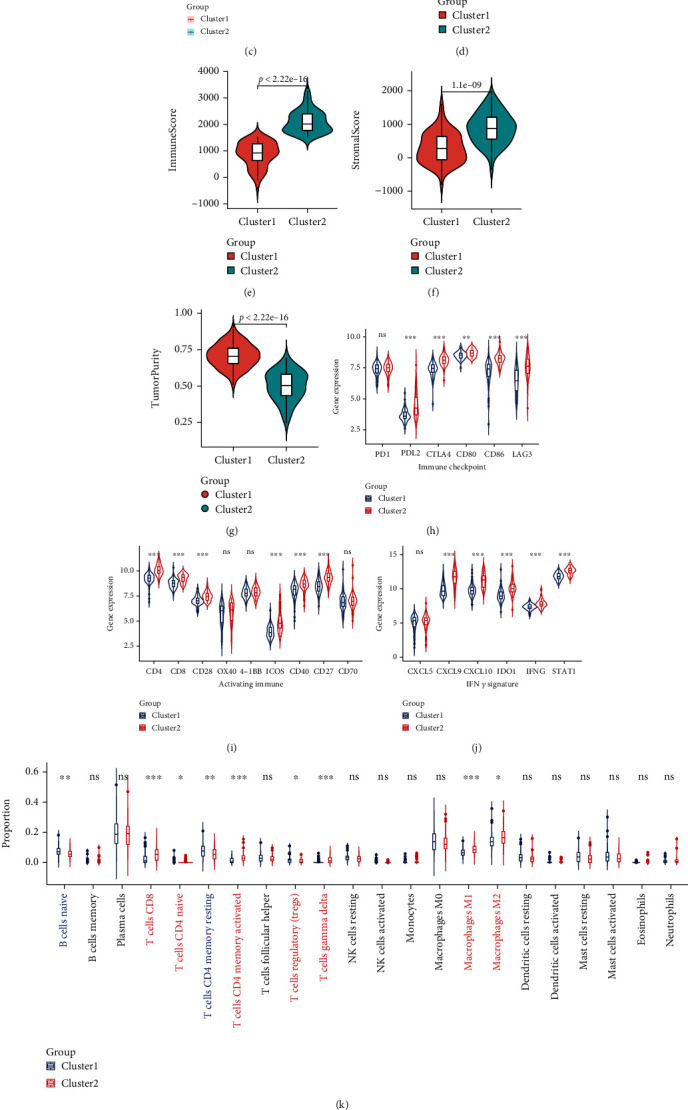
GEO validation of PTGIS and HRASLS in evaluating prognosis and tumor immunity of LUSC. (a) Based on the expression of PTGIS and HRASLS, the GSE4753 cohort was divided into two clusters. (b) Association between PTGIS and HRASLS expression in the GSE4753 cohort. (c) The Kaplan-Meier survival curve of two clusters. Comparison of ESTIMATEScore (d), ImmuneScore (e), StromalScore (f), TumorPurity (g), the expression of immunomodulatory markers (h–j), proportion of 22 immune cells (k), and expression of 28 immune cells (l) between two clusters. ns: no significance. ^∗^*p* < 0.05, ^∗∗^*p* < 0.01, and ^∗∗∗^*p* < 0.00.

We divided the GSE4573 cohort into two clusters using the same consensus clustering method as the TCGA cohort and found that the expression distribution of PTGIS and HRASLS in the two clusters was similar to that in the TCGA cohort (Figure 13(a)). The comparison of clinicopathological data showed that the pN and pTNM stages of cluster 1 were higher than that of cluster 2 (Table 4). The Spearman correlation analysis showed that the expression levels of PTGIS and HRASLS were negatively correlated, which was similar to the TCGA cohort (*r* = −0.21, *p* = 0.018) (Figure 13(b)). Survival analysis showed that high expression of PTGIS and low expression of HRASLS were associated with poor prognosis in LUSC (Supplementary Figure 6), and the OS rate of cluster 2 was significantly higher than that of cluster 1 (Figure 13(c)). We used the same analysis method as the TCGA cohort to compare the degree of tumor immune infiltration including ESTIMATE (Figures 13(d)–13(g)), the expression of immunomodulatory markers (Figures 13(h)–13(j)), CIBERSORT (Figure 13(k)), and ssGSEA (Figure 13(l)) between the two clusters. Unsurprisingly, the immune system of cluster 2 was more active.

## 4. Discussion

Due to the lack of specific clinical manifestations and diagnostic biomarkers in the early stage of LUSC, most of the patients were diagnosed with advanced tumors with distant metastasis, resulting in high morbidity and mortality of LUSC [1, 37]. The use of immunotherapy has revolutionized the treatment of patients with advanced LUSC, but determining accurate individualized treatment is a difficult problem [12]. Therefore, it is of great clinical significance to determine reliable biomarkers for the efficacy and prognosis of immunotherapy in LUSC, contributing to the designation of accurate clinical decision-making of individual patients' immunotherapy.

Metabolic reprogramming, including changes in lipid metabolism, was considered to be a characteristic manifestation of tumors. Lipid metabolism reprogramming of the TME affected tumor cell growth, proliferation, invasion, metastasis, and immune escape [38, 39]. Multigene models of tumor physiological and pathological pathways played an important role in predicting tumor clinical prognosis and outcome [40]. Gene signatures of glucose metabolism [41], lipid metabolism [28], and amino acid metabolism [42] pathways have been reported to play an important role in predicting tumor prognosis and immune characteristics. However, the role of LMRGs in LUSC has not been reported.

In our study, we determined that PTGIS and HRASLS were associated with LUSC prognosis and immunity and divided the TCGA cohort into two clusters based on their consensus clustering. Prostaglandin I2 synthase (PTGIS) was a member of the cytochrome P450 superfamily and encoded a monooxygenase involved in lipid syntheses such as cholesterol and steroids. PTGIS was a key gene that catalyzed the transformation of prostaglandin H2 to prostaglandin I2 (PGI2) [43]. PGI2 was an important product of the arachidonic acid (AA) metabolic pathway and an important immunomodulatory lipid mediator, which affected the normal inflammatory response and the activation and differentiation of immune cells [44, 45]. PTGIS was highly expressed in colon cancer tissues and liver metastases and was associated with liver metastasis and poor prognosis of colon cancer [46]. Dai et al. [47] have shown that PTGIS could be used as a potential biomarker for prognosis and tumor immune infiltration of lung, ovarian, and gastric cancers. The sequence of the H-RAS-like suppressor (HRASLS) was homologous to lecithin: retinol acyltransferase (LRAT) [48]. All members of the HRASLS family could metabolize phospholipids in vitro and participate in a wide range of biological processes [49, 50], but the biological activity in vivo has not been fully studied.

The important role of PTGIS and HRASLS in LUSC was further clarified. In vitro, we found that PTGIS was a tumor-promoting gene of LUSC and HRASLS was a tumor-suppressor gene. The results of consensus clustering showed that the expression of PTGIS and HRASLS had the opposite trend in the two clusters. Then, we performed GSEA on both clusters and found that all DEGs were enriched in immune activation regulatory pathways, such as activation of the immune response, acute inflammatory response, and adaptive immune response. Therefore, we speculated that cluster 2 may have stronger immune infiltration than cluster 1.

The immune infiltration in the TME was related to the clinical features and prognosis of NSCLC [51, 52]. For example, M1 macrophages [53], CD4 cells, CD8 cells [54, 55], NK cells [56], and dendritic cells [57] in NSCLC promoted antitumor immune responses and improved prognosis; M2 macrophages [58], regulatory T cells [59, 60], and Th17 cells [61] were associated with immunosuppression and poor prognosis in NSCLC. ICI treatment works by enhancing the body's natural antitumor response, and a stronger antitumor immune response in individuals may make it easier for patients to benefit clinically from ICI treatment [62]. Our study showed that the infiltrating proportion of immune-activated cells, expression of immune checkpoints, and TMB level in cluster 2 were significantly higher than those in cluster 1, and cluster 2 had a better prognosis. Therefore, LUSC patients in cluster 2 were more likely to benefit from immunotherapy.

The changes in tumor lipid metabolism provided a new therapeutic target for tumor therapy. At present, preclinical and clinical studies have shown that multiple targeted lipid metabolism schemes showed good antitumor effects [13]. Our study suggested that PTGIS and HRASLS played an important role in LUSC phenotype, prognosis, and tumor immunity. In conclusion, PTGIS and HRASLS can be used as new biomarkers and therapeutic targets for LUSC and have positive significance in guiding the immunotherapy of LUSC.

There were still some limitations in this study. Firstly, this study was a retrospective study based on public databases (TCGA and GEO databases). Therefore, prospective studies based on the real world should be carried out in the future to increase the clinical value of the research. In addition, the potential value of PTGIS/HRASLS response to LUSC immunotherapy has not been verified in clinical samples. This study was based on transcriptome data and did not prove the direct mechanism of PTGIS/HRASLS in LUSC immune infiltration and antitumor immunity.

## 5. Conclusion

We identified LMRGs PTGIS and HRASLS that were related to the prognosis and tumor immunity of LUSC. In vitro, PTGIS and HRASLS affected LUSC proliferation, migration, and invasion. Based on consensus clustering of PTGIS and HRASLS expression distribution, LUSC patients in TCGA and GSE4573 cohorts were divided into two clusters. Cluster 2 had a better prognosis, stronger immune infiltration, higher expression of immune checkpoints, and higher TMB levels than cluster 1. Therefore, our study suggests that PTGIS and HRASLS have potential clinical value in guiding immunotherapy as novel therapeutic targets for LUSC as well as biomarkers for prognosis and tumor immunity.

## Figures and Tables

**Table 1:** Characteristics of LUSC patients in the training set and validation set.

	TCGA-LUSC cohort (*n* = 394)	GSE4573 cohort (*n* = 130)	Statistical value	*p* value
Age (median [IQR])	68 ([62, 73])	68 ([60, 75])	-0.35^a^	0.726
Gender (%)			6.426	0.011^∗^
Female	100 (25.4)	48 (36.9)		
Male	294 (74.6)	82 (63.1)		
pT stage (%)			0.301	0.583
1-2	322 (81.7)	109 (83.8)		
3-4	72 (18.3)	21 (16.2)		
pN stage (%)			0.007	0.935
N0	250 (63.5)	83 (63.8)		
N1-N3	144 (36.5)	191 (36.2)		
pTNM stage (%)			0.163	0.687
I-II	318 (80.7)	107 (82.3)		
III-IV	76 (19.3)	23 (17.7)		

^a^Mann–Whitney *U* test. ^∗^*p* < 0.05.

**Table 2:** The siRNA sequences used in RNA interference analysis.

Gene	Sense (5′⟶3′)	Antisense (5′⟶3′)
PTGIS-siRNA1	CGGUGACAUCUUUACUAUACU	UAUAGUAAAGAUGUCACCGUG
PTGIS-siRNA2	CACAAAUGCUAUUCAGAUAAG	UAUCUGAAUAGCAUUUGUGGA
PTGIS-siNC	UUCUCCGAACGUGUCACGUTT	ACGUGACACGUUCGGAGAATT
HRASLS-siRNA1	GGUGUAUUACAGACCAGAACC	UUCUGGUCUGUAAUACACCGG
HRASLS-siRNA2	CAUACAGAAUAAACAAUAAAU	UUAUUGUUUAUUCUGUAUGUG
HRASLS-siNC	UUCUCCGAACGUGUCACGUTT	ACGUGACACGUUCGGAGAATT

**Table 3:** Clinical characteristics of two clusters in the TCGA-LUSC cohort.

	Cluster 1 (*n* = 199)	Cluster 2 (*n* = 195)	Statistical value	*p* value
Age (median [IQR])	68 ([60, 73])	69 ([62, 74])	-1.720	0.086
Gender (%)			1.089	0.297
Female	46 (23.1)	54 (27.7)		
Male	153 (76.9)	141 (72.3)		
pT stage (%)			3.963	0.047^∗^
T1-2	155 (77.9)	167 (85.6)		
T3-4	44 (22.1)	28 (14.4)		
pN stage (%)			25.787	0^∗∗∗^
N0	102 (51.3)	148 (75.9)		
N1-N3	97 (48.7)	47 (24.1)		
pM stage			2.626^a^	0.215
M0	194 (97.5)	194 (99.5)		
M1	5 (2.5)	1 (0.5)		
pTNM stage (%)			15.9	0^∗∗∗^
I-II	145 (72.9)	173 (88.7)		
III-IV	54 (27.1)	22 (11.3)		

^a^Fisher exact probability test. ^∗^*p* < 0.05 and ^∗∗∗^*p* < 0.001.

**Table 4:** Clinical characteristics of two clusters in the GSE4573 cohort.

	Cluster 1 (*n* = 79)	Cluster 2 (*n* = 51)	Statistical value	*p* value
Age (median [IQR])	68 ([59, 75])	66 ([61, 75])	-0.634	0.528
Gender (%)			0.625	0.419
Female	27 (34.2)	21 (41.2)		
Male	52 (65.8)	30 (58.8)		
pT stage (%)			2.498	0.114
T1-2	63 (79.7)	46 (90.2)		
T3-4	16 (20.3)	5 (9.8)		
pN stage (%)			4.134	0.042^∗^
N0	45 (57)	38 (74.5)		
N1-N3	34 (43)	13 (25.5)		
pTNM stage (%)			5.591	0.018^∗^
I-II	60 (75.9)	47 (92.2)		
III-IV	19 (24.1)	4 (7.8)		

^∗^ *p* < 0.05.

## Data Availability

Publicly available datasets were used in this study. These data can be found in three databases: The Cancer Genome Atlas (TCGA), the Gene Expression Overview (GEO), and the Genotype-Tissue Expression (GTEx). The rest of the datasets used and analyzed during the current study are available from the corresponding authors on reasonable request.

## Abbreviations

AA: Arachidonic acid
ALK: Anaplastic lymphoma kinase
DEGs: Differentially expressed genes
ECL: Enhanced chemiluminescence
EdU: 5-Ethynyl-2′-deoxyuridine
FASN: Fatty acid synthetase
GEO: Gene Expression Omnibus database
GO: Gene Ontology
GS: Gene significance
ICIs: Immune checkpoint inhibitors
IHC: Immunohistochemistry
IOD: Integral optical density
KEGG: Kyoto Encyclopedia of Genes and Genomes
LMRGs: Lipid metabolism-related genes
LRAT: Lecithin: retinol acyltransferase
LUSC: Lung squamous cell carcinoma
MM: Module membership
NSCLC: Non-small-cell lung cancer
OS: Overall survival
PPI: Protein-protein interaction
SCLC: Small cell lung cancer
SDS-PAGE: Sodium dodecyl sulfate-polyacrylamide gel electrophoresis
SNP: Single nucleotide polymorphism
SNV: Simple nucleotide variation
siRNAs: Short interfering RNAs
ssGSEA: Single-sample gene set enrichment analysis
TCGA: The Cancer Genome Atlas
TMB: Tumor mutation burden
WB: Western blot
WGCNA: Weighted gene coexpression network analysis.

## Supplementary Materials

Supplementary Materials Supplementary Table 1: lipid metabolism gene set in Reactome pathway database. Supplementary Table 2: the mRNA expression matrix of lipid metabolism-related genes (LMRGs) in the TCGA TARGET GTEx dataset. Supplementary Table 3: the mRNA expression matrix of lipid metabolism-related genes (LMRGs) in the TCGA dataset. Supplementary Table 4: the mRNA expression matrix of the GSE4573 cohort. Supplementary Figure 1: the expression of key genes in lipid uptake pathway in LUSC and normal lung tissues. ^∗∗∗^*p* < 0.001. Supplementary Figure 2: (A) the Kaplan-Meier survival curve of ALDH3B1. (B) The Kaplan-Meier survival curve of CSNK2A1. (C) The Kaplan-Meier survival curve of LCLAT1. Supplementary Figure 3: the TCGA cohort's heatmap corresponds to the consensus matrix for *k* = 2 using consensus clustering. Supplementary Figure 4: the infiltration heatmap of 22 kinds of immune cells in two clusters of the TCGA cohort. Supplementary Figure 5: sample clustering of the TCGA cohort to detect outliers (height = 60). Supplementary Figure 6: (A) the Kaplan-Meier survival curve of PTGIS in the GSE4573 cohort. (B) The Kaplan-Meier survival curve of HRASLS in the GSE4573 cohort.
