# Correction to “Effects of Lipid Metabolism‐Related Genes PTGIS and HRASLS on Phenotype, Prognosis, and Tumor Immunity in Lung Squamous Cell Carcinoma”

**DOI:** 10.1155/omcl/9853912

**Published:** 2026-06-01

K. Lei, R. Liang, B. Tan, et al., “Effects of Lipid Metabolism‐Related Genes PTGIS and HRASLS on Phenotype, Prognosis, and Tumor Immunity in Lung Squamous Cell Carcinoma,” *Oxidative Medicine and Cellular Longevity*, 2023, 6811625, https://doi.org/10.1155/2023/6811625.

In the article, an error was identified by the authors in Figure 7, that was introduced during the figure assembly process. Specifically, the Migration/si‐2 panel in Figure 7c is erroneously duplicated in the Migration/Vector panel of Figure 7f. The correct Figure 7 is shown below:

Figure 7HRASLS inhibits LUSC proliferation, migration, and invasion. (a) The mRNA expression of HRASLS in the SK‐MES‐1 cell line transfected with siRNAs or si‐NC was measured by qRT‐PCR. (d) The overexpression plasmid of HRASLS or the control vector was transfected in the SK‐MES‐1 cell line, and the mRNA expression of HRASLS was measured by qRT‐PCR. (b, e) Representative images of EdU assay after HRASLS knockout (b) and HRASLS overexpression (e) in SK‐MES‐1 cells. (c, f) Representative images of transwell assay after HRASLS knockout (c) and HRASLS overexpression (f) in SK‐MES‐1 cells.  ^∗∗∗^ *p* < 0.001.

**Figure:**
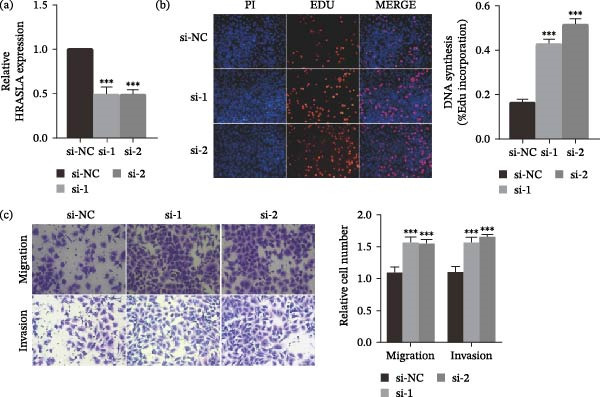

**Figure:**
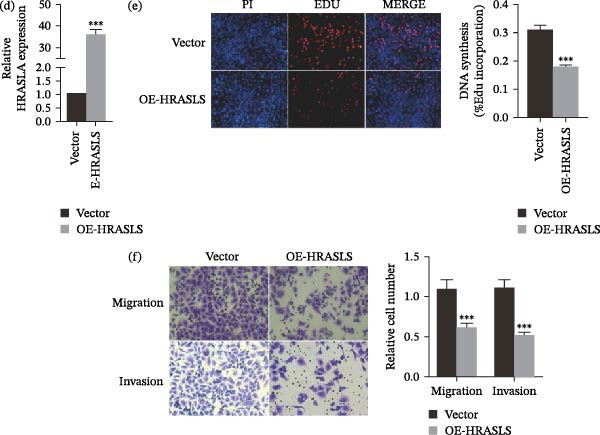

We apologize for this error.

